# Tumor- and mitochondria-targeted nanoparticles eradicate drug resistant lung cancer through mitochondrial pathway of apoptosis

**DOI:** 10.1186/s12951-019-0562-3

**Published:** 2020-01-09

**Authors:** He Wang, Fangke Zhang, Huaying Wen, Wenwen Shi, Qiudi Huang, Yugang Huang, Jiacui Xie, Peiyin Li, Jianhai Chen, Linghao Qin, Yi Zhou

**Affiliations:** 1 Key Laboratory of Molecular Clinical Pharmacology & Fifth Affiliated Hospital, Guangzhou Medical University, Guangzhou, 511436 Guangdong China; 2 Center of Cancer Research, The Second Affiliated Hospital, Guangzhou Medical University, Guangzhou, 510260 Guangdong China; 3 School of Pharmacy, Guangdong Pharmaceutical University, Guangzhou, 510006 Guangdong China; 4 Nanfang Hospital, Southern Medical University, Guangzhou, 510515 Guangdong China

**Keywords:** Mitochondrial targeting, Paclitaxel, Nanomicelles, Multidrug resistance, Cancer therapy

## Abstract

Chemotherapeutic drugs frequently encounter multidrug resistance. ATP from mitochondria helps overexpression of drug efflux pumps to induce multidrug resistance, so mitochondrial delivery as a means of “repurposing’’ chemotherapeutic drugs currently used in the clinic appears to be a worthwhile strategy to pursue for the development of new anti-drug-resistant cancer agents. TPP-Pluronic F127-hyaluronic acid (HA) (TPH), with a mitochondria-targeting triphenylphosphine (TPP) head group, was first synthesized through ester bond formation. Paclitaxel (PTX)-loaded TPH (TPH/PTX) nanomicelles exhibited excellent physical properties and significantly inhibited A549/ADR cells. After TPH/PTX nanomicelles entered acidic lysosomes through macropinocytosis, the positively charged TP/PTX nanomicelles that resulted from degradation of HA by hyaluronidase (HAase) in acidic lysosomes were exposed and completed lysosomal escape at 12 h, finally localizing to mitochondria over a period of 24 h in A549/ADR cells. Subsequently, TPH/PTX caused mitochondrial outer membrane permeabilization (MOMP) by inhibiting antiapoptotic Bcl-2, leading to cytochrome C release and activation of caspase-3 and caspase-9. In an A549/ADR xenograft tumor model and a drug-resistant breast cancer-bearing mouse model with lung metastasis, TPH/PTX nanomicelles exhibited obvious tumor targeting and significant antitumor efficacy. This work presents the potential of a single, nontoxic nanoparticle (NP) platform for mitochondria-targeted delivery of therapeutics for diverse drug-resistant cancers.

## Introduction

Lung cancer is the leading cause of cancer-related mortality worldwide [1]. Despite recent advances in treatment, lung cancer remains an incurable disease [2]. Paclitaxel (PTX), which is a natural plant product extracted from the bark of western yew (*Taxus brevifolia*), has commonly been used as a promising front-line agent for the treatment of lung cancer and exhibits activity against a broad range of cancers mainly by acting on the mitochondria [3–5] and microtubules of cancer cells [6]. However, the anticancer activity of PTX is significantly limited due to its poor aqueous solubility [7]. Taxol is a pharmaceutical formulation of PTX and is used in the clinical treatment of cancers. However, Taxol causes a severe hypersensitivity reaction due to the solvent Cremophor EL in its formulation [6]. However, multidrug resistance, whether inherent or acquired, has dramatically compromised the effectiveness of drug efflux pumps toward PTX [8].

Although chemotherapy plays a primary role in the management of cancers, the efficacy of chemotherapy seems to be decreased by the multidrug resistance of cancers. Mitochondria are the powerhouses of the cell and serve as attractive targets for cancer treatment. Multidrug-resistant (MDR) cancer cells exhibit increased mitochondrial mass with more polarized mitochondria than non-MDR cells [9]. As multidrug resistance arises due to the overexpression of drug efflux pumps, which require ATP from mitochondria, mitochondrial targeting is a particularly sensible option for the treatment of drug-resistant cancer cells [10, 11]. Thus, in MDR cancer cells, the highly polarized mitochondrial membranes are important targets and are associated with ATP-dependent drug efflux.

Delocalized lipophilic cations play a key role in mitochondrial targeting [12], accumulating to a greater degree in the mitochondria of cancer cells than in those of normal cells due to the high negative mitochondrial membrane potentials of cancer cells [13]. Triphenylphosphonium (TPP) is frequently used in delocalized lipophilic cations, which usually decorate the surfaces of nanoparticles (NPs) or are covalently linked to nanocarriers for mitochondrial targeting [13]. Furthermore, good treatment results were acquired. However, single targeting of mitochondria is difficult to deal with the increasing drug resistance of tumor [14].

In order to further play a role which TPP targeted mitochondrial, PF127 (FDA approved, poly(ethylene oxide)-block-poly(propylene oxide)-block-poly(ethylene oxide), PEO-PPO-PEO triblock copolymer) was applied into modify TPP, has been widely used as a pharmaceutical adjuvant. Moreover, this polymer can interact with cell membranes, leading to decreased microviscosity, pore formation on the membrane and accelerated “flip-flop” of the membrane component, increasing the reversion of drug resistance. However, PF127, with a high hydrophile–lipophile balance (HLB) value, exhibits poor cellular membrane binding [15]. It is hypothesized that the conjugation of TPP with PF127 (TPP-PF127, TP) could decrease the HLB value. Thus, TP would be easily internalized into tumor cells.

**Scheme 1:**
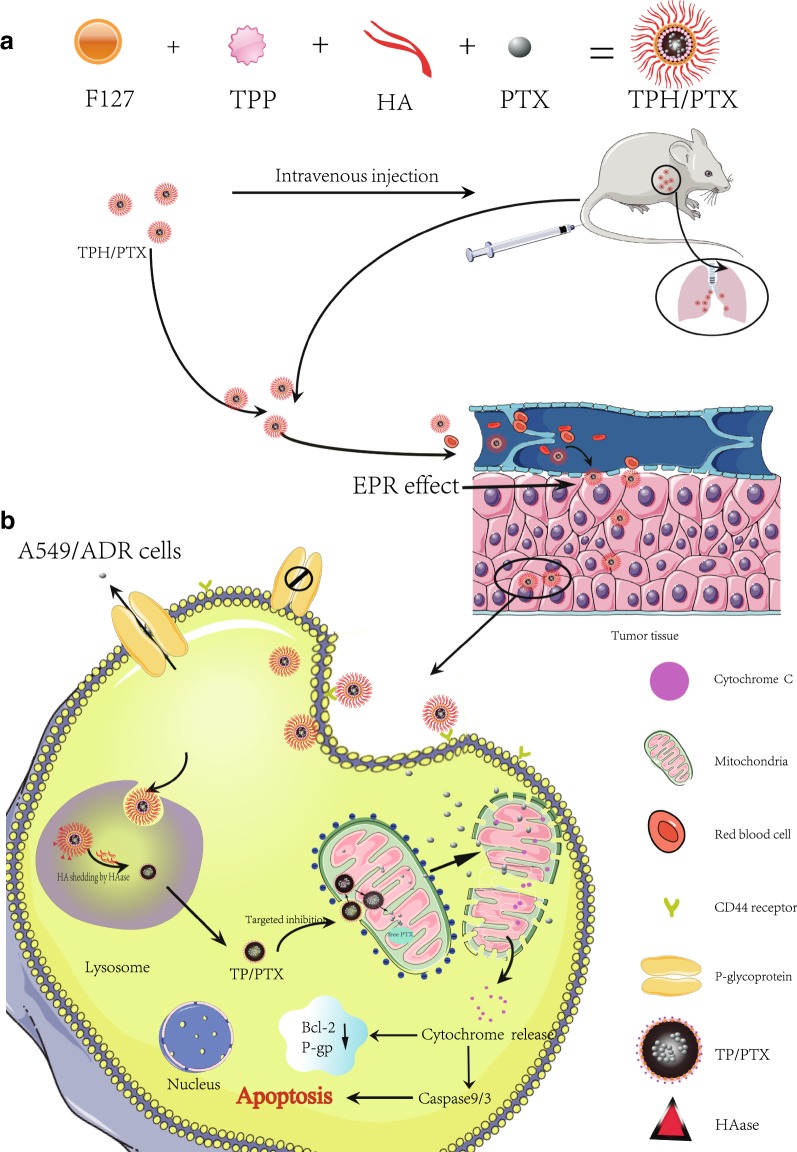
**a** Schematic representation of the assembly of TPH/PTX nanomicelles. **b** The transporting pathway in vivo of TPH/PTX nanomicelles

To neutralize the positive charges of TP nanomicelles, to avoid quick clearance and to achieve long-term circulation, negatively charged hyaluronic acid (HA) was further grafted with OH-PF127-TPP through covalent bonds due to the hydrophilic and negatively charged outer shell [16]. Simultaneously, specific tumor-targeting nanomicelles were found between TPP-PF127-HA (TPH) and CD44 receptors overexpressed on tumor cells. As illustrated in Scheme 1, PTX-loaded TPH NPs will target tumor cells through ligand-receptor interactions. The HA molecules could be degraded by hyaluronidase (HAase), which is highly abundant in the tumor extracellular matrix and lysosomes. The lysosome escape of nanomicelles depends on the positive charges and proton sponge effect of the quaternary ammonium groups of TP nanomicelles [17]. Finally, positively charged TP/PTX nanomicelles accumulate in negatively charged mitochondria and induce MDR cancer cell apoptosis by activating intrinsic mitochondrial apoptosis pathways.

## Methods

### Materials and general characterization

Pluronic F127 (Mw = 12,600) was purchased from BASF aktiengesellschaft, and (5-carboxypentyl)triphenylphosphonium bromide (TPP-COOH) was purchased from Bailingwei Co., Ltd., Guangzhou, China. HA and HAase were acquired from J&K Scientific Ltd. (Beijing, China). *N*,*N*′-dicyclohexylcarbodiimide (DCC), 4-dimethylaminopyridine (DMAP), genistein, chlorpromazine, amiloride, coumarine-6 (C6), and PTX were purchased from Sigma-Aldrich (Shanghai, China). Fetal bovine serum (FBS) and RPMI 1640 medium were purchased from HyClone and Gibco, respectively (Thermo Fisher Scientific Co., Ltd., Shanghai, China). The near-infrared (NIR) fluorescent dye DIR was supplied by Keygen Biotech (KGMP0026, China). Hematoxylin and eosin (H&E) staining reagents were supplied by Leagene Biology Technology Co., Ltd. (Beijing, China). MitoTracker Red and LysoTracker^®^ Red were purchased from Yeasen Biotech Co., Ltd. (Shanghai, China). All other reagents were of analytical grade.

### Synthesis of TPP-PF127-HA

#### Synthesis of the PF127-TPP copolymer

The TPP-PF127 (TP) conjugate was synthesized by the procedures reported by Kun Na [18, 19]. Briefly, PF127 powder (100 mg, 1 mmol) was dissolved in anhydrous DMSO. COOH-TPP (9.1 mg, 2.5 mmol), DCC (1.5 mmol), and DMAP (1.5 mmol) were dissolved in DMSO (5 mL). The reaction mixture was stirred at room temperature using a magnetic stirrer and allowed to react under nitrogen for 24 h, followed by dialysis (Mw cut-off: 1000 Da) for 3 days against distilled water to remove unconjugated COOH-TPP and DMSO. The final solution was flash-frozen dry and lyophilized.

#### Synthesis of the TPP-PF127-HA copolymer

HA and TP were dissolved in DMSO (3 mL) in a molar ratio of 2:1 with large amounts of DCC and DMAP. The reaction mixture was stirred at room temperature using a magnetic stirrer and allowed to react under closed conditions for 24 h. The filtrate was dialyzed for 24 h (Mw cut-off: 10,000 Da) and dried under reduced pressure.

### Preparation of PTX-loaded TPP-PF127-HA (TPH/PTX) nanomicelles

PTX-loaded nanomicelles were prepared using a nanoprecipitation method. Briefly, nanoprecipitation involved the dissolution of TPH in water to form an aqueous phase, while PTX was dissolved in an organic solvent such as acetone or ethanol to form an oil phase. Then, the oil phase was injected into the water phase under stirring; stirring was continued; and the organic solvent was evaporated. Finally, the drug-loaded micelles were obtained, and the spherical or spheroidal micelles were observed by transmission electron microscopy (TEM). TP/PTX was prepared using the same procedure. The particle size and zeta potential of the NPs were characterized using a Malvern Zetasizer Nano ZS90. In vitro TPH/PTX drug release was measured by HPLC (Waters Corp., Waltham, MA, USA). The prepared TPH/PTX nanomicelles were suspended in phosphate-buffered saline (PBS, 0.02 M, pH 7.4) containing PTX at a concentration of 50 μg/mL. After the solution (1 mL) was transferred to dialysis tubing, 0.2% Tween PBS (30 mL) was added to immerse the tubing. At predetermined intervals, the external buffer was collected, and an equivalent volume of fresh buffer was added. The concentration of PTX in the collected solution was determined by HPLC analysis [20]. The drug loading capacity (DLC) and drug loading efficiency (DLE) were calculated according to the following formulas:

$$ {\text{DLC }}\left( \% \right)  = {\text{ weight of drug used}}/\left( {{\text{weight of polymer }} + {\text{ drug used}}} \right) \times { 1}00\% $$

$$ {\text{DLE }}\left( \% \right)  = {\text{weight of loaded drug}}/{\text{weight of input drug }} \times { 1}00\% $$

### Stability

The stability of the TPH in different conditions was examined using the protocol described in reference [21].

### Cell culture

Human lung adenocarcinoma A549 cells, drug-resistant A549 cells overexpressing P-gp (A549/ADR), and drug-resistant mouse breast cancer 4T1 (4T1/ADR) cells (College of Pharmaceutical Science, Guangzhou Medical University, Guangzhou, China) were grown in RPMI-1640 supplemented with 10% FBS and 1% antibiotics (100 U/mL penicillin and 100 mg/mL streptomycin). For maintenance of drug resistance, A549/ADR and 4T1/ADR cells were cultured in the presence of 4 µM CDDP, and CDDP-free medium was used for 1 week prior to initiation of the experiments [20]. Cell cultivation was performed in a humidified incubator maintained at 37 °C containing 5% CO_2_. All of the cell studies were approved by the Institutional Animal Care Committee and the Local Veterinary Office and Ethics Committee at Guangzhou Medical University (GZMUC 10-05010).

### Cytotoxicity and hemo-compatibility of nanomicells

A549 cells or A549/ADR cells were incubated with fresh culture media containing varying concentrations of TP, TPH, Taxol, TP/PTX, and TPH/PTX nanomicelles at 37 °C for 1 h. The final concentration of PTX was approximately 0–50 μM, and the concentration of the blank nanomicelles was consistent with that of the PTX-loaded nanomicelles. Blank culture medium was used as the blank control. After 24 h, 48 h, and 72 h of incubation, cell viability was measured by proliferation assays [3], with each assay performed in triplicate. Finally, dose–effect curves were created, and the drug concentration that inhibited 50% of cell growth (IC 50) was calculated by curve fitting the cell viability data to that of the control samples.

Hemo-compatibility of nanomicelles is examined using the protocol described in reference [22].

### Apoptosis-inducing effect

Apoptosis rates were measured using the FITC Annexin V-staining Kit and a FACScan flow cytometer (BD, USA). A549 and A549/ADR cells were seeded in six-well plates (5 × 10^5^ cells/well). After incubation for 24 h, the medium was replaced with RPMI 1640 supplemented with the above formulations with 10 μM PTX. After 24 h of incubation, cell apoptosis was detected using the FITC Annexin V-staining Kit and a FACScan flow cytometer according to the standard protocol.

### Investigation of the endocytosis mechanism by CLSM

A total of 5 × 10^4^ A549/ADR cells were seeded on a cover slip for 12 h, followed by washing with PBS. The cells were preincubated with 1 mM 5-(*N*-ethyl-*N*-isopropyl)-amiloride, 10 µg/mL chlorpromazine, and 200 µM genistein for 30 min. Control cells were treated without inhibitors, followed by incubation with TPH/C6 (10 μg/mL) nanomicelles at 37 °C for 2 h. The nuclei of the cells were further labeled with 2 µg/mL DAPI for 10 min. After incubation, the A549/ADR cells were imaged by confocal laser scanning microscopy (CLSM) (Zeiss LSM 710).

### Lysosome escape

CLSM was used for the lysosome escape assay. A549 and A549/ADR seeded in a special confocal microscopy dish (NEST) at a density of 5 × 10^4^ cells/well, respectively. After 24 h, Taxol, TP/C6, and TPH/C6 nanomicelles were added to the media and incubated. At predetermined time intervals (2 h, 4 h, 6 h, and 8 h), the cells were washed with cold PBS and then stained with 1 μM LysoTracker Red for 30 min and 2 µg/mL DAPI for 10 min at 37 °C in the dark. Subsequently, the cells were washed and observed by CLSM.

### Mitochondria localization

A549 and A549/ADR cells were seeded in a Lab-Tek 8-well chamber slide at 1.5 × 10^4^ cells per well. After 12 h of attachment, cells were incubated with TPH/C6 for 6 h, 12 h and 24 h. Mitochondria were labeled with 1 μM MitoTracker Red and imaged by CLSM.

### Drug content in the isolated mitochondria

FACScan flow cytometer was applied into drug content in isolated mitochondria assay. A549 and A549/ADR cells, were seeded in 6-well plates at a density of 2 × 10^5^ cells/well for 48 h, were treated by PBS, Taxol, TP/PTX, TPH/PTX nanomicells containing 10 µM of PTX for 24 h. The cells were then collected and washed by cold PBS twice. Mitochondria isolation was performed according to mitochondria isolation kit protocols (Beyotime Institute of Biotechnology, China). The amount of C6 in mitochondrial was analyzed by FACScan flow cytometer and indicated by fluorescent intensity. The assay was repeated in triplicate.

### JC1 assay

A549 and A549/ADR cells were seeded in a Lab-Tek 8-well chamber slide. Taxol, TP/C6, and TPH/C6 nanomicelles were incubated with 1.5 × 10^4^ cells for 24 h. A control was included without the NPs. The cells were then washed twice with PBS (pH = 7.4) and incubated with 10 µg/mL 5,5′,6,6′-tetrachloro-1,1′,3,3′-tetraethylbenzimidazolylcarbocyanine iodide (JC1) dye (Beyotime Institute of Biotechnology, China) at 37 °C for 20 min. Before adding JC1 dye, the cells were visualized using CLSM at a fluorescence emission wave length of 590 nm, and the red fluorescence signal obtained from the NPs was calculated to be deducted from the JC1 fluorescence emission signal for baseline correction. The JC1 signal in the cells was visualized and quantified by CLSM.

### Release of cytochrome C

The release of cytochrome C from the mitochondria of A549 and A549/ADR cells into the cytosol was measured using a streptavidin-peroxidase immunohistochemical kit (Zhongshan Goldenbridge Biotechnology, Co., Ltd., Beijing, China) [23]. Briefly, after incubation for 24 h, A549 and A549/ADR cells were exposed to Taxol, TP/C6, and TPH/C6 nanomicelles or fresh medium as a control. The cells were then fixed with paraformaldehyde for 20 min and sequentially treated with Triton x-100, 3% H_2_O_2_, and the provided blocking reagent. Next, the cells were incubated with primary antibody overnight at 4 °C. Then, the secondary antibody (provided in the kit) and the enhanced streptavidin HRP conjugate (provided in the kit) were added to the cells. After color development, the release of cytochrome C was observed under a light microscope.

### Caspase activation

A549 and A549/ADR cells were cultured for 12 h and then treated with Taxol, TP/PTX, and TPH/PTX nanomicelles. Controls samples were prepared by adding blank medium. The final concentration of PTX was 10 µM. After 12 h of incubation, the cells were harvested, lysed, and analyzed by Western blotting. The following antibodies were used: anti-caspase-9, anti-caspase-3, Bcl-2, and Bax (all from Cell Signaling, Beverly, MA, USA) [24].

### Xenograft tumor model establishment and biodistribution analyses

4T1/ADR-bearing mice BALB/C mice (5 weeks old, weighing 16–18 g) were cultured in our Lab. All animal experiments were carried out in compliance with the guidelines of the Institutional Animal Care and Use Committee of Guangzhou Medical University. For establishment of the subcutaneous xenograft tumor models, 4T1/ADR cells (3 × 10^6^) were administered by subcutaneous injection into the right flanks of the mice. When the volumes of the tumors reached approximately 200–300 mm^3^, the mice were administered physiological saline, free DIR and DIR-loaded nanomicelles via tail vein injection. At predetermined time points (2 h, 6 h, 12 h and 24 h), images were obtained with an NIR fluorescence imaging system. The mice were sacrificed by dislocation of the cervical vertebra 24 h after injection. Then, the tumor and organs, including the heart, liver, spleen, lung and kidney, were collected and analyzed in the imaging system.

### Antitumor efficacy in vivo

Twenty tumor-bearing mice (A549/ADR) were used when the volume of the tumors reached approximately 220–230 mm^3^. The animals were randomly divided into four groups, namely, groups treated with saline, Taxol, TP/PTX, and TPH/PTX nanomicelles (n = 5), and treated at days 17, 19, 21, 23, 25, 27, and 29 via the tail vein. The final concentration of PTX was 10 mg/kg. The mice were then monitored with respect to tumor progression and weight loss every other day, and the tumor volumes were calculated as the length × width^2^/2 (mm^3^). The tumor volume inhibition rate at day 29 was calculated using the formula Rv = 100% − (V drug /V saline) × 100%, where V drug is the tumor volume after drug treatment, and V saline is the tumor volume after treatment with physiological saline. H&E staining assays were further performed. Moreover, an immunohistochemical assay of the tumor tissue was also performed to evaluate the release of cytochrome C. H&E stained to examine the tissue toxicity of the therapeutic agents.

### Antitumor effects on lung metastasis in the drug-resistant breast cancer-bearing mouse models

Twenty-five BALB/C nude mice (female, 6–8 weeks old) were divided into five groups (five mice per group). On day 0, all of the mice were administered 1 million 4T1/ADR tumor cells by intravenous injection to generate a breast cancer-bearing mouse model with lung metastasis. At 6, 8, 10, 13 and 15 days, the four groups were treated by systemic administration of PBS, Taxol, TP/PTX nanomicelle, and TPH/PTX nanomicelle. The final concentration of PTX was 10 mg/kg. On day 15, the mice were sacrificed, and the light signal and tumor number from the lung was then immediately captured and counted to further validate tumor growth, respectively.

### Statistical analysis

Statistical analyses were performed using GraphPad Prism 5.0 software. Comparisons were statistically assessed by one-way ANOVA. The data are presented as the mean ± standard deviation (SDs).

## Results

### Synthesis and characterization of TPH/PTX

**Scheme 2:**
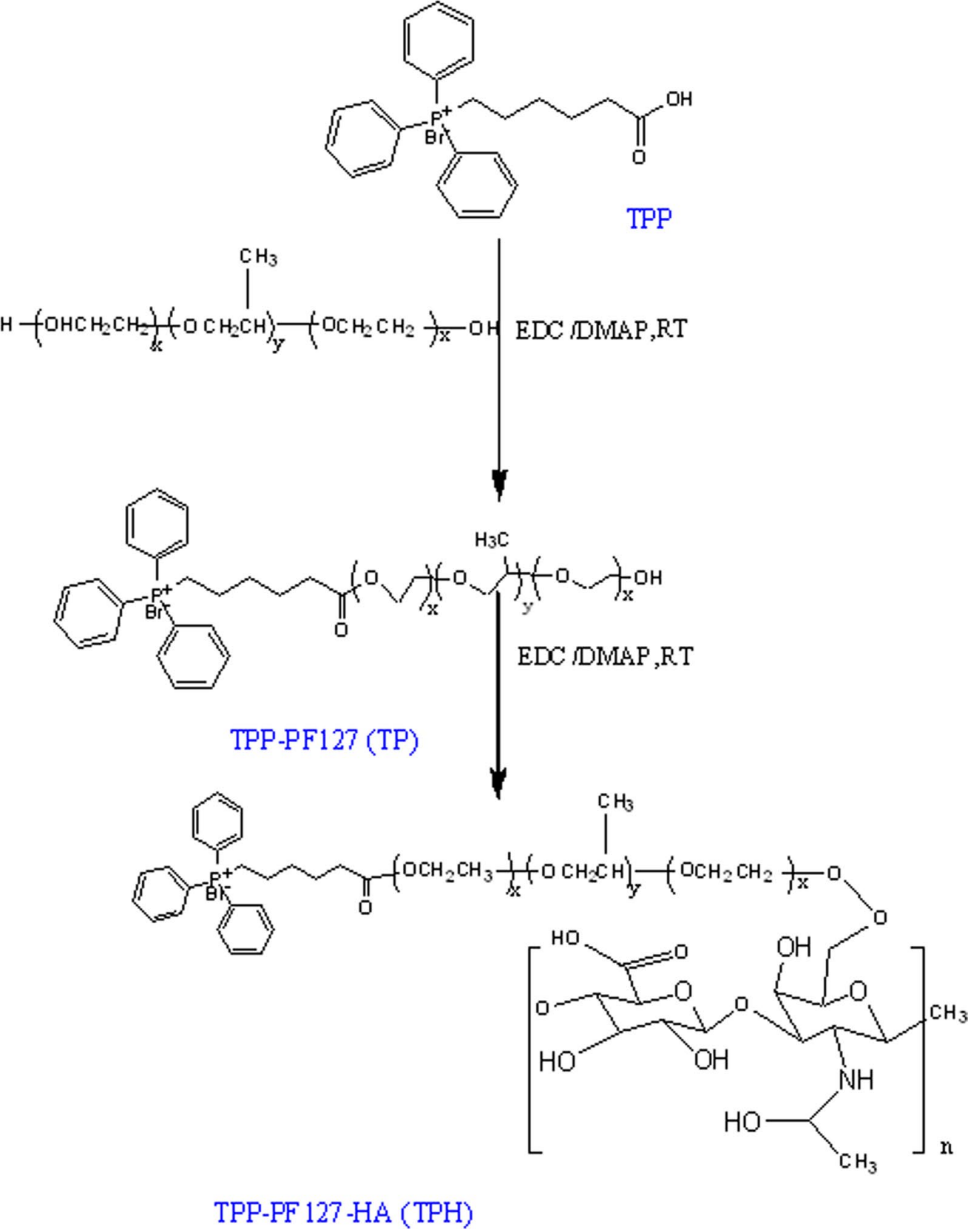
Synthetic scheme showing the various steps required to prepare TPH

**Fig. 1:**
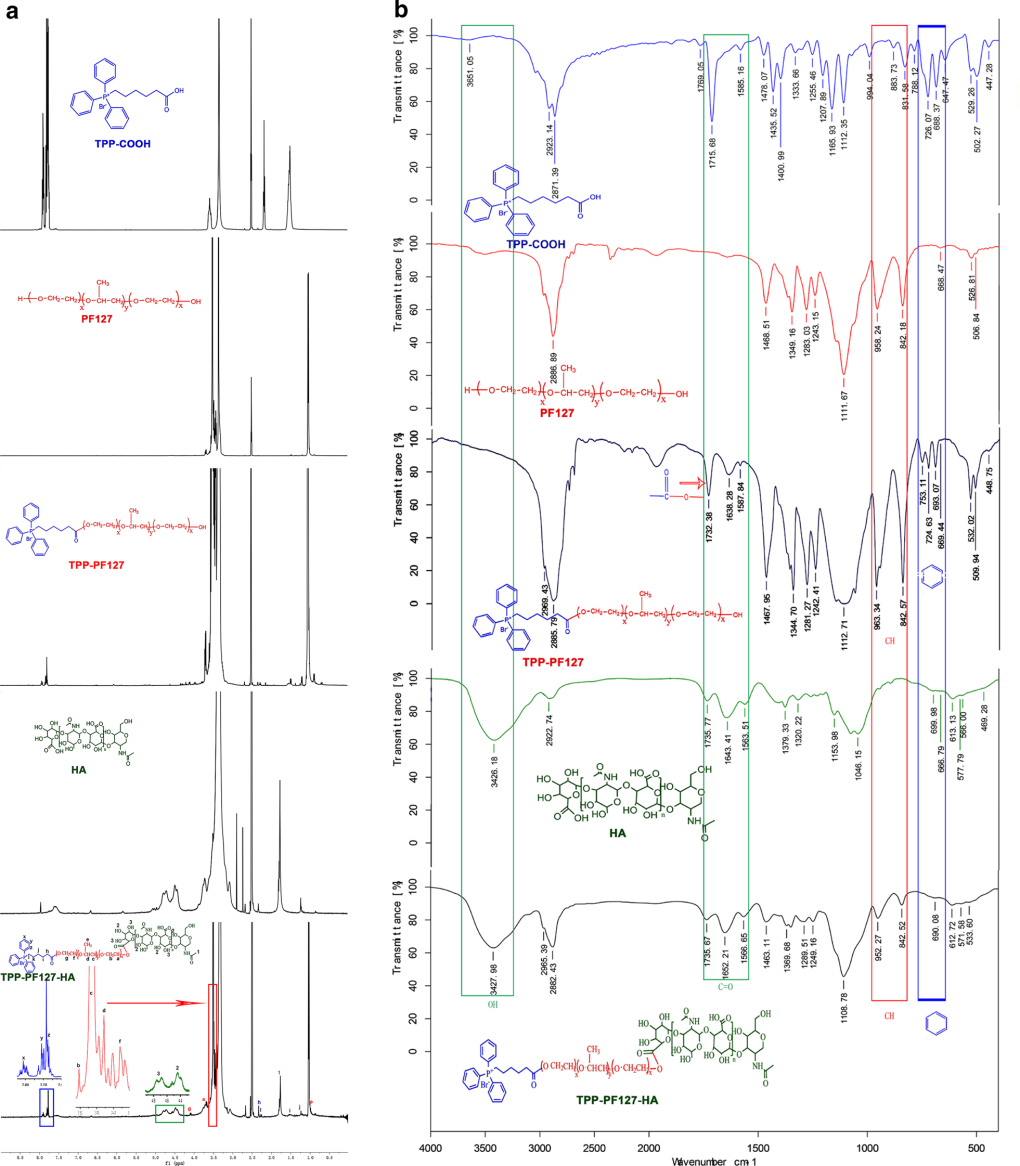
^1^H NMR (**a**) and IR (**b**) of PF127, TPP, HA, TP, and TPH polymers

Recently, it was demonstrated that TPP, as part of the vectors, can transfer chemotherapeutic drugs into cancer cells and simultaneously target mitochondria in cancer cells [25]. In this study, F127 was conjugated to COOH-TPP by esterification using DCC/DMAP. This reaction produced OH-PF127-TPP (TP). The average diameter of TP/PTX nanomicelles was approximately 135 nm, with a positive zeta potential of + 13.2 mV. To better overcome the drug resistance of cancer cells and reduce the high positive zeta potential, HA was conjugated to TP by esterification using DCC/DMAP. HA-PF127-TPP (TPH) was finally produced. The average diameter of the TPH nanomicelles was approximately 142 nm, which was approximately 7 nm higher than that of the TP/PTX nanomicelles, with a negative potential of − 24.65 mV (Table 1). The route used for the synthesis of TPH is shown in Scheme 2. The ^1^H-NMR and IR spectrum complete peak assignments of the TPH copolymer were showed in Fig. 1a, b, respectively.

**Table 1:** Characterization of nanomicelles

Formulation	Particle size (nm)	PDI	Zeta potential	Encapsulation (%)
Blank nanomicelles	126 ± 9.56	0.254 ± 0.015	− 27.36 ± 3.65	
TP/PTX nanomicelles	135 ± 8.65	0.281 ± 0.020	13.24 ± 3.45	90.51 ± 9.23
TPH/PTX nanomicelles	142 ± 8.35	0.235 ± 0.020	− 24.65 ± 2.75	92.35 ± 9.05

**Fig. 2:**
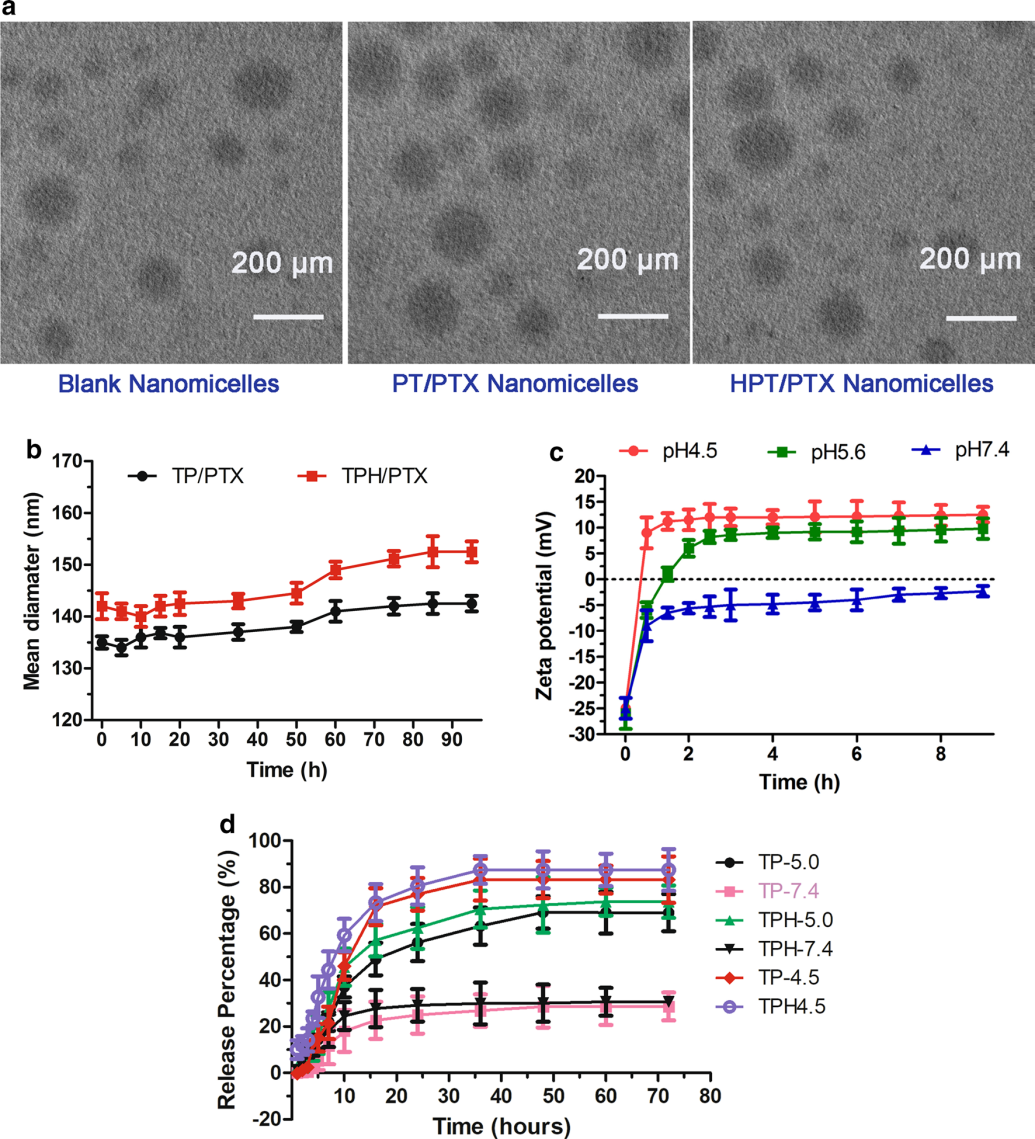
Characterization of all nanomicells. **a** Representative TEM images of all nanomicells. **b** Size change of all nanomicells after incubation in PBS containing 10% FBS at 37 °C. **c** PTX’s release from all nanomicells at pH 7.4. **d** Change in the zeta potential of TPH/PTX nanomicells after incubation with HAase (0.5 mg/mL) at different pH. Data are presented as the mean ± SD (n = 3)

The TEM assay showed that all the nanomicelles were spherical (Fig. 2a). The diameter of the TPH/PTX nanomicelles was slightly increased compared with those of the TP/PTX and blank nanomicelles, indicating successful modification of HA on TP.

To investigate the stability of TPH/PTX, TP/PTX and TPH/PTX nanomicelles were added to PBS (pH 7.4) containing 10% FBS at 37 °C. The particle size showed no significant change over 96 h (Fig. 2b), implying a strong negative potential and that the hydrophilic HA shell protected TP/PTX from opsonization by plasma proteins for further enhancement of stability. The zeta potential of TPH/PTX, however, rapidly increased when the nanomicelles were exposed to HAase with obvious pH dependence; the values were + 9.48 mV at pH 5.6 and + 12.85 mV at pH 4.5, from − 4.58 mV at pH 7.4, after a 10-h incubation, implying that TPH/PTX nanomicelles escaped from the acid lysosome through the proton sponge (Fig. 2c).

As shown in Fig. 2d, the release percentage of PTX at 10 h from TPH/PTX nanomicelles was approximately 30% at pH 7.4. However, at pH 5.0, the cumulative release of PTX from TPH/PTX was increased to 68% at 72 h, and at pH 4.5, the cumulative release increased to 83% at 72 h, indicating that the release profiles are dependent on the acid concentration.TP/PTX nanomicelles are similar to TPH/PTX, remaining approximately unchanged for 72 h and exhibiting a markedly prolonged release.

### In vitro antitumor activity

**Fig. 3:**
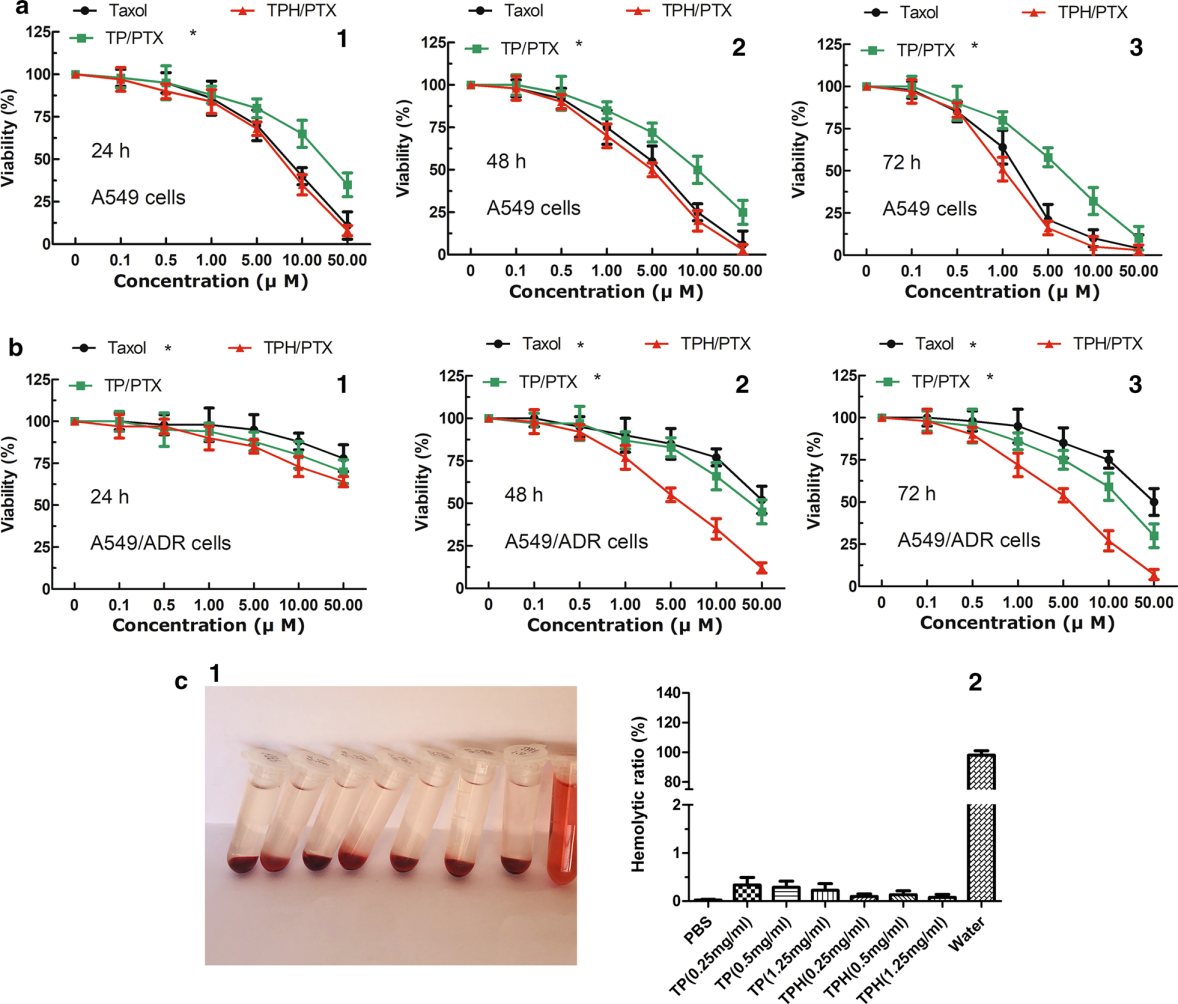
Antitumor efficacy in vivo. Viability of A549 (**a**) and A549/ADR (**b**) cells cultured with PTX-loaded nanomicelles in comparison with that of Taxol at the same PTX dose for 24 h (1), 48 h (2), and 72 h (3), respectively. All data are presented as the means ± standard deviations (n = 3); It is hemolytic ratio (**c**) and corresponding photographs (**d**) of mice erythrocytes that have been treated differently. From left to right: PBS, TP (0.25 mg/mL, 0.5 mg/mL, 1.25 mg/mL), TPH (0.25 mg/mL, 0.5 mg/mL, 1.25 mg/mL), and water

To understand the effect of the NP-mediated mitochondrial damage in cancer cells, A549 and A549/ADR cells were treated with TPH/PTX nanomicelles for 24 h, 48 h, and 72 h, followed by evaluation of cell viability by proliferation assays. As shown in Fig. 3a, cancer cell viability decreased with increasing PTX concentrations and extended incubation times. TP/PTX nanomicelles showed lower anti-A549 cell activity than Taxol at all doses, but TP/PTX nanomicelles showed greater inhibition of A549/ADR than Taxol alone (Fig. 3b). Compared with Taxol and TP/PTX nanomicelles, TPH/PTX showed high antitumor activity with extended duration of incubation (24 h, 48 h and 72 h). In particular, for A549/ADR cancer cells, TPH/PTX exhibited much lower IC 50 values (35.25, 16.41, and 9.66 μM) than Taxol (70.48, 61.52, and 58.53 µM) and TP/PTX nanomicelles (47.39, 42.87, and 32.38 µM) at 24 h, 48 h, and 72 h (Table 2), which might be attributed to the efficient internalization of nanomicelles with HA serving as the active targeting ligand that specifically binds CD44 receptors overexpressed in many tumor cells [26] and mitochondrial target.

**Table 2:** IC50 values of Taxol and every PTX loaded nanomicelles on A549 and A549/PTX cells after 24 h, 48 h, and 72 h incubation (n = 5)

Formulation	A549			A549/ADR		
	Taxol	TP/PTX	TPH/PTX	Taxol	TP/PTX	TPH/PTX
24 h	18.64 ± 0.53	36.54 ± 2.31	14.79 ± 1.56	70.48 ± 2.86	47.39 ± 3.25	35.25 ± 2.54
48 h	9.44 ± 1.32	28.22 ± 2.15	6.68 ± 1.23	61.52 ± 2.30	42.87 ± 2.36	16.41 ± 1.35
72 h	4.71 ± 0.45	14.91 ± 1.65	3.07 ± 0.45	58.53 ± 1.32	32.38 ± 3.12	9.66 ± 1.25

The in vitro biocompatibility of TP and TPH polymer was further studied by performing the hemo-compatibility test (Fig. 3c). The hemolysis levels of TP and TPH nanomicelles were negligible with hemolytic ratios of 0.001 vitro biocompatibility of TP and TPH polymer was further studied by performing the hemo-cafter co-cultured with PBS, TP (0.25 mg/mL, 0.5 mg/mL, 1.25 mg/mL), TPH (0.25 mg/mL, 0.5 mg/mL, 1.25 mg/ml), respectively. In addition, the cytotoxicity of drug free nanomicelles (for example, TP, TPH), were also assayed in lung cancer cells, and cell cytotoxicity was hardly observed, indicating that these vectors are biocompatible and non-toxic to tissues and cells (Additional file 1: Figure S1).

### Cellular uptake

**Fig. 4:**
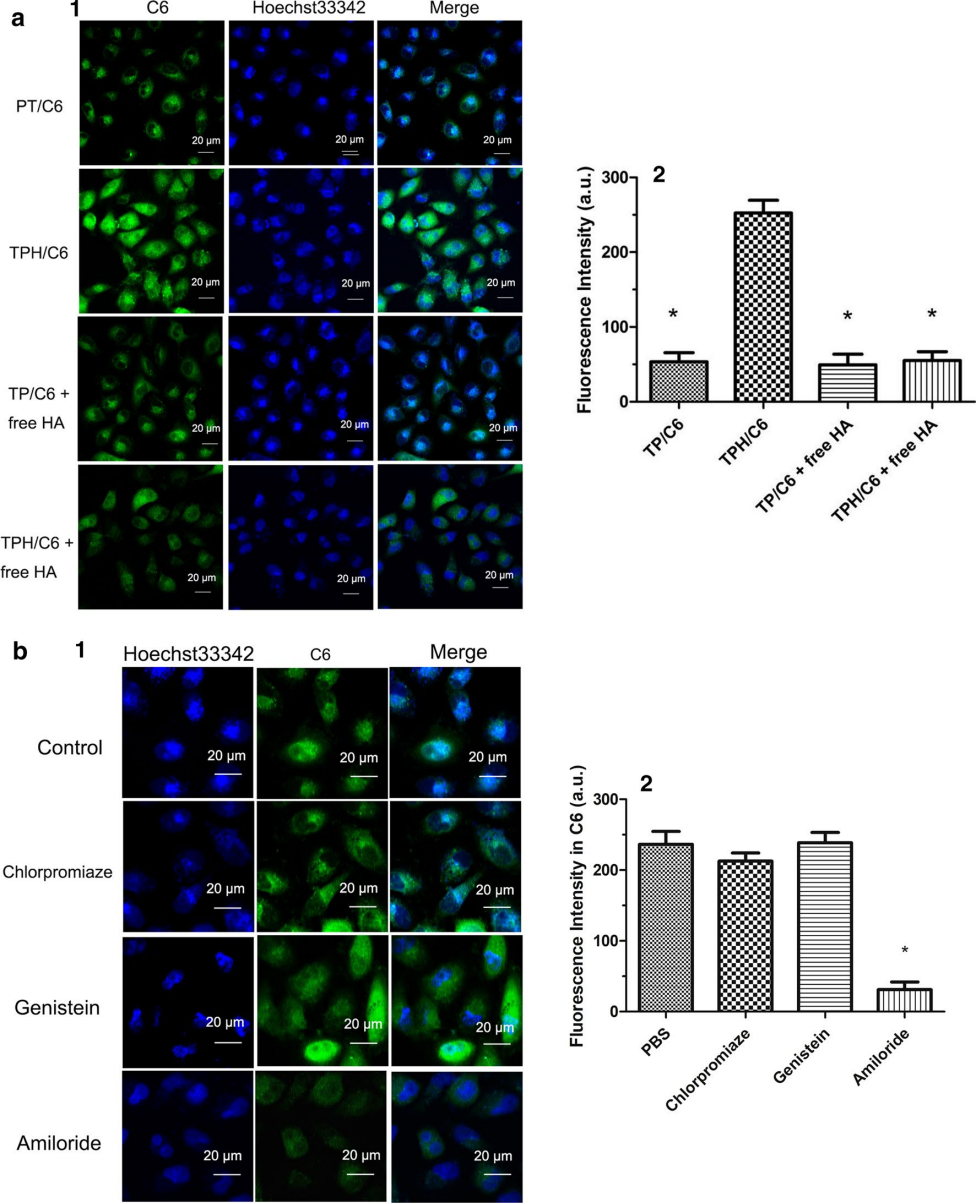

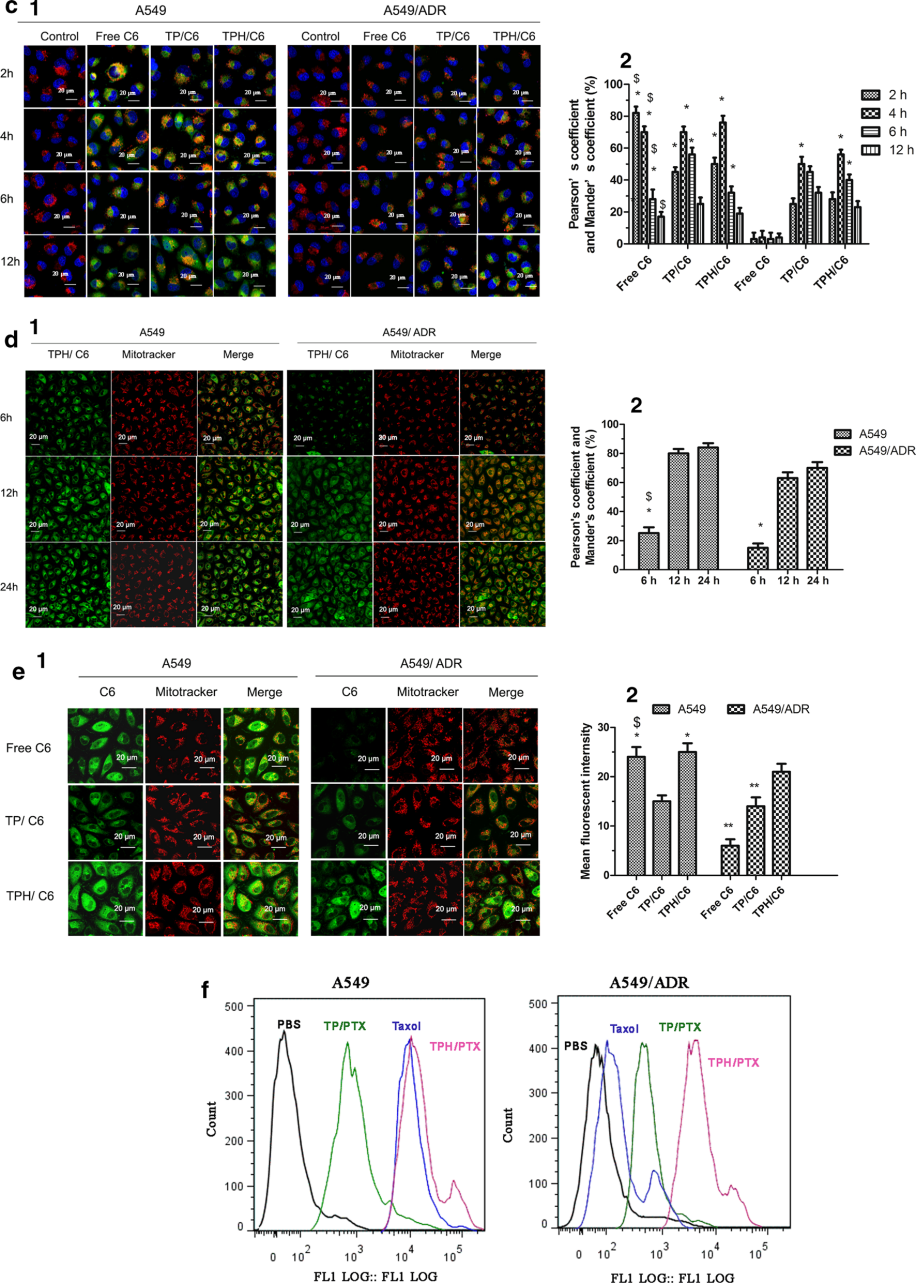
**a** A CLSM images of A549/ADR cells after 30 min of incubation with TP/C6 (non-targeted) and TPH/C6 (targeted). *P < 0.05, vs. TPH/C6. **b** A CLSM images of A549/ADR cells pre-treated with chlorpromazine, genistein and amiloride followed by treatment with TPH/C6 nanomicells. Nucleus were stained by hoechst33342 (blue) dyes. *P < 0.05, vs. PBS. **c** Merge CLSM image showed lysosomes escape of every nanomicells on A549 and A549/ADR cells. The late endosomes and lysosomes were stained by LysoTracker Red. Green fluorescence indicate C6. Yellow fluorescence indicated the overlay between C6 and lysosomes. Blue fluorescence indicated hoechst33342. *P < 0.05, vs. 12 h; ^$^*P* < 0.05, vs. free C6 in A549/ADR. **d** CLSM images of A549 and A549/ADR cells showing the homing of HTP/C6 nanomicells into mitochondria for 6 h, 12 h and 24 h time points. Mitochondria were stained with MitoTracker Red dyes. The yellow regions in the merged images indicate the co-localization of HTP/C6 nanomicells in mitochondria. **e** Co-localization of the Taxol, TP/C6, TPH/C6 into mitochondria at A549 and A549/ADR cells at 24 h observed by CLSM. The mitochondria were stained by MitoTracker Red. C6 emits green fluorescence itself. Yellow fluorescence indicates the overlay between C6 and MitoTracker Red. *^,^**P < 0.05, vs. TP/C6 nanomicells. ^$^*P* < 0.05, vs. free C6. **f** Drug contents determined in the isolated mitochondrial fraction isolated from A549 cells by flow cytometry. *P < 0.05, vs. 12 h or 24 h; ^$^*P* < 0.05, vs. 6 h in A549/ADR, respectively. (1) Image of CLSM; (2) fluorescence intensity of image. Scale bars are 20 μm

CLSM can be used to study the cellular uptake of nanomicelles. As shown in Fig. 4a, the fluorescence intensity of TPH/C6 increased 4.8-fold compared with that of the nontargeted TP/C6 nanomicelles in the HA-free group. However, when TPH/C6 was added to A549/ADR cells that contained saturated HA, the cellular uptake efficiency of the targeted TPH/C6 decreased substantially due to the competitive binding of free HA and CD44 on the cancer cells, suggesting that the endocytosis of TPH/C6 nanomicelles by A549/ADR cells was greatly facilitated by CD44-mediated internalization.

The highly hydrophobic free C6 readily diffused into the A549 cells and drastically induced cellular accumulation. However, the fluorescence intensity of C6 changed weakly in the resistant A549/ADR cells. Interestingly, the C6 green fluorescence of TPH/C6 nanomicelles was more intense than that of TP/C6 nanomicelles or free C6 in both A549 and A549/ADR cells. Importantly, the intense green fluorescence of TPH/C6 in A549/ADR cells was similar to that in A549 cells, indicating that the tumor-targeting and mitochondria-targeting TPH/PTX nanomicelles can overcome drug resistance.

### Mechanism of endocytosis

To investigate the endocytosis machinery involved, A549/ADR cells preincubated with different endocytosis inhibitors (genistein, chlorpromazine and amiloride) were treated with TPH/C6 nanomicelles for 2 h. As shown in Fig. 4b, genistein- and chlorpromazine-pretreated cells endocytosed TPH/C6 nanomicelles to the same extent as non-inhibitor-treated control cells. On the other hand, the amiloride complex exhibited the most significant inhibitory effect among the three endocytic inhibitors, indicating that macropinocytosis-mediated endocytosis was the main pathway for endocytosis of TPH/C6 nanomicelles.

### Lysosome escape

The TPH/PTX nanomicelles must escape lysosomes before being trafficked to mitochondria after accumulation in the acidic lysosomes. Further investigation of the mechanism underlying lysosome escape in A549/ADR cells showed that colocalization of the red fluorescence of lysosomes and green fluorescence of C6 led to a merged yellow signal at different time points. Figure 4c demonstrates that all nanomicelles exhibited obvious merged signals (weak red, strong yellow, weak yellow, and strong green) at 2 h, 4 h, 6 h, and 12 h, respectively. Compared with other groups, TPH/C6 was mainly accumulated in lysosomes at 4 h and separated from the lysosomes and completely distributed in the cytoplasm at 12 h. Although lysosomal sequestration of the drug pumped out A549/ADR cells led to the lose of large amounts of free C6, the fluorescence intensity of TPH/C6 in the cytoplasm of A549/ADR cells approached that in A549 cells, suggesting that most TPH/C6 can successfully escape lysosome in 12 h.

### Colocalization in the mitochondria

After escaping from lysosomes, TPH/C6 nanomicelles should be trafficked to mitochondria to deliver their payloads. To observe the localization in mitochondria, TPH/C6 nanomicelles were incubated with A549 and A549/ADR cells for three different durations (6 h, 12 h and 24 h), followed by staining of the mitochondria with MitoTracker red dye. The merged yellow signal, which came from the green fluorescent TPH/C6 nanomicelles colocalized with red fluorescent-tagged mitochondria, was imaged by CLSM, as shown in Fig. 4d. Pearson’s coefficient and Mander’s coefficient-based quantification of the volume of colocalization demonstrated 80.5% and 84.2% (A549) and 63.5% and 70% (A549/ADR) overlapping regions at 12 h and 24 h, respectively, which were more yellow than the signals observed at 6 h (25.2%, A549; 16.8%, A549/ADR), moreover, the merged light yellow signal from TPH/C6 nanomicells significantly was showed, compared with TP/C6 nanomicells (Fig. 4e), indicating that TPH/C6 nanomicelles localized in mitochondria over 24 h.

To further confirm the localization of TPH/C6 in mitochondria, all nanomicelles were incubated with A549/ADR cells for 24 h. Flow cytometry assays demonstrated that the mean fluorescence intensity of the isolated mitochondria in TPH/C6 nanomicelles was the highest compared to that of free C6 and TP/C6 nanomicelles (Fig. 4f). The result further verified the accumulation of the nanomicelles in mitochondria, which supported the mitochondrial colocalization observed by CLSM.

### Mitochondrial outer membrane permeabilization.

**Fig. 5:**
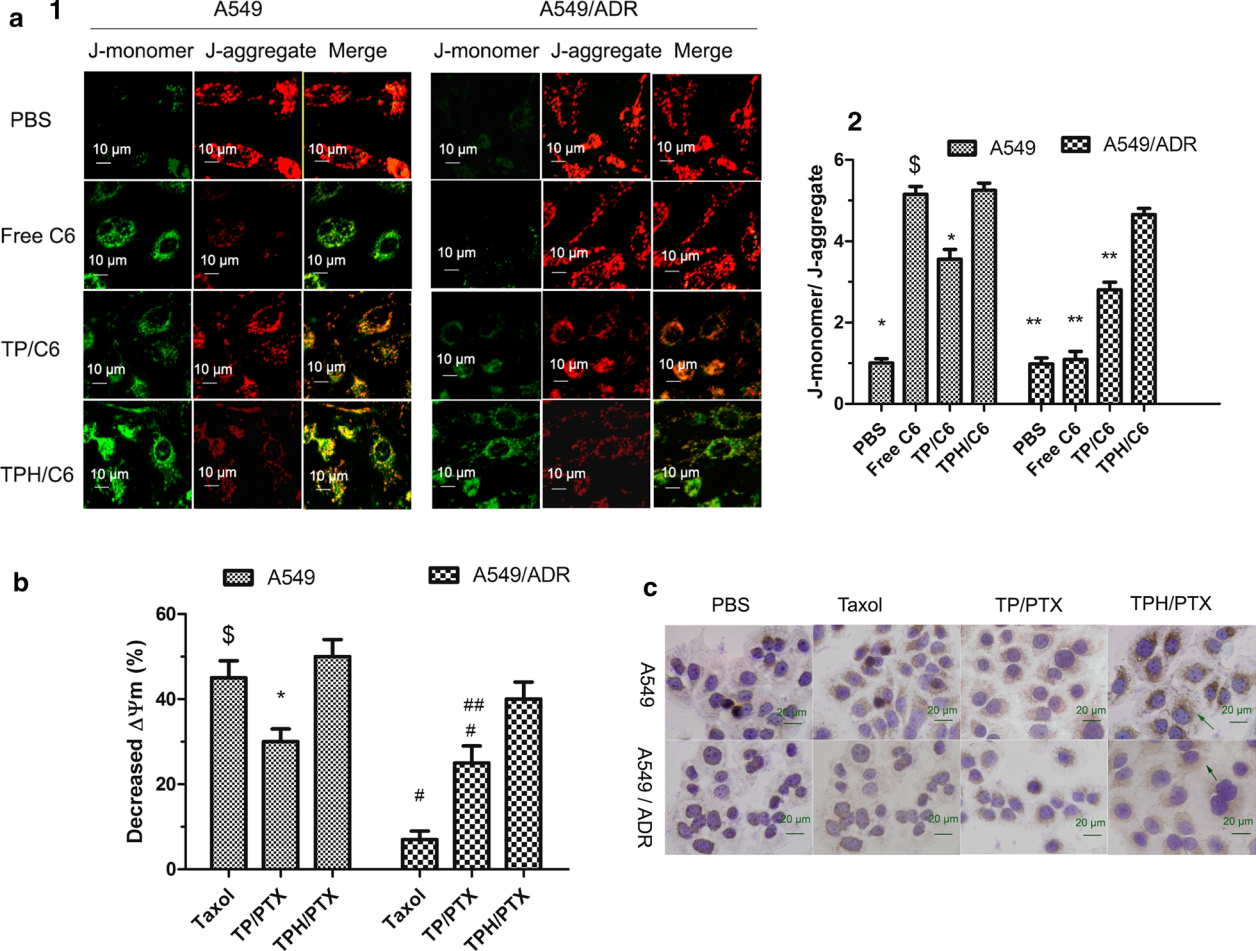
**a** Mitochondrial depolarization of A549 and A549/ADR cells treated with TPH/C6 followed by JC1 dye was observed by CLSM. The green and red colors were generated by JC1 dye in monomeric and aggregated forms respectively. **b** Immunohistochemical staining images of cytochrome C translocated from mitochondria to cytosol in the A549 and A549/ADR cells after applying PBS, Taxol, TP/PTX, and TPH/PTX. Data are presented as the mean ± SD (n = 3). *P < 0.05, ^#^*P* < 0.05 vs. TPH/PTX nanomicells; ^$^*P* < 0.001 vs. Taxol in A549/ADR. ^##^*P* < 0.05 vs. Taxol. (1) Image of CLSM; (2) Fluorescence intensity of image

After trafficking into mitochondria and successful release of drugs, JC1 was used to investigate the effect of TPH/PTX nanomicelles on the mitochondrial membrane potential (Δψm). JC-1, as a lipophilic cationic dye, can selectively swarm into mitochondria and reversibly change the red signal to green when the mitochondrial membrane potential is reduced. The strong green fluorescence indicated a decrease in Δψm. Figure 5a demonstrates that the green/red ratios induced by PBS, Taxol, TP/PTX nanomicelles, and TPH/PTX nanomicelles in A549/ADR cells were 0.98 ± 0.07, 1.09 ± 0.05, 2.85 ± 0.10, 4.65 ± 0.11, respectively. The values were similar to those observed in A549 cells (1.01 ± 0.07, 5.15, 3.56 ± 0.11, 5.25 ± 0.04). By comparison, the decrease of Δψm of TPH/PTX nanomicells was the most significant, which was almost 4.26-times higher than that of the Taxol group in A549/ADR (Fig. 5b).

Mitochondrial outer membrane permeabilization (MOMP) leads to the release of cytochrome C from the intermembrane space (IMS) [23]. The release of cytochrome C from mitochondria was observed by optical microscopy using a streptavidin-peroxidase immunohistochemical kit. As shown in Fig. 5c, compared to the control, Taxol and TP/PTX nanomicelles exhibited release of small amounts of cytochrome C (brown). The highest release in A549 and A549/ADR cells was observed with TPH/PTX nanomicelles, which was consistent with the results of the mitochondrial membrane potential and cell apoptosis experiments. The release of cytochrome C may be related to the opening of the MPTP (mitochondrial permeability transition pore) as the direct effect of the PTX molecules or the activation of the proapoptotic protein Bax [27].

### In vitro apoptosis-inducing effect

**Fig. 6:**
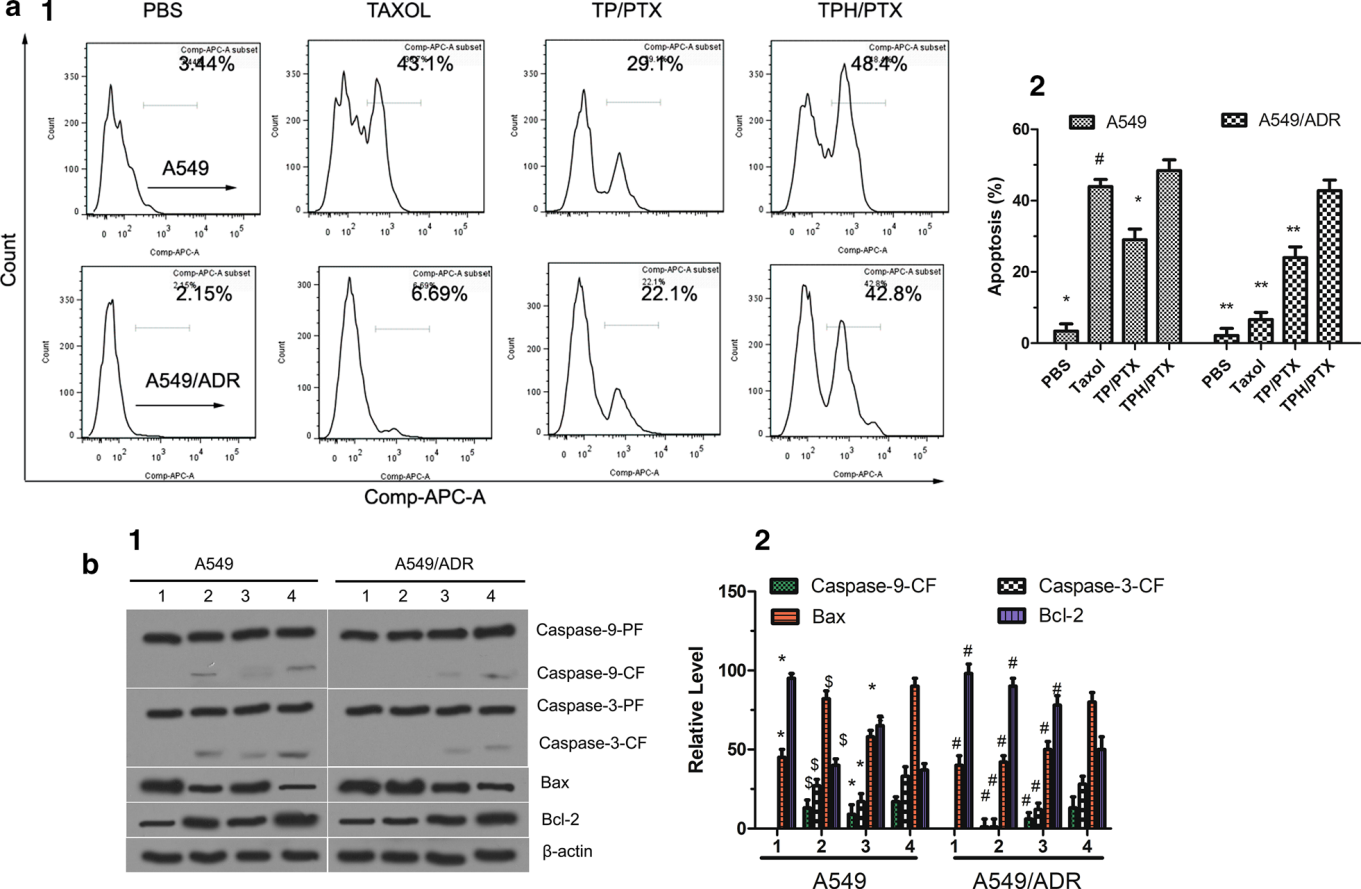
**a** Cell apoptosis rate detected by flow cytometry. A549 and A549/ADR cells were treated with different formulations that contained a total PTX concentration of 10 μM for 24 h. **b** Proteins involved in the apoptosis signaling pathways in A549 and A549/ADR cells as determined by Western blotting. ① Control (PBS); ② Taxol; ③ TP/PTX; and ④ TPH/PTX nanomicells. Activity ratios of caspase-3 and caspase-9 and expression ratios of the pro-apoptotic proteins Bax and the anti-apoptotic proteins Bcl-2 in A549 and A549/ADR cells after incubation with the various formulations. β-actin was also assessed by Western blotting. All protein levels were quantified densitometrically and normalized to β-actin. All data are presented as the means ± standard deviations (n = 3); (1) Image of western blot; (2) Grey level of western blot. *P < 0.05, compared with TPH/PTX nanomicells. ^#^P < 0.05, compared with Taxol in A549 cells

The Annexin V-FITC Apoptosis Detection Kit was used to test whether TPH/PTX nanomicelles can induce apoptosis in drug-resistant cells. Figure 6a depicts the apoptosis-inducing effects of all the nanomicelles in A549 and A549/ADR cells. After the addition of PBS, Taxol, TP/PTX nanomicelles, and TPH/PTX nanomicelles, the apoptosis rates in A549/ADR cells were 2.15, 6.69, 22.10, and 42.80%, respectively, while those in A549 cells were 3.44, 43.10, 29.1, and 48.40%. These findings further indicated that TPH/PTX nanomicelles could effectively overcome drug resistance.

### Apoptosis-related signaling pathways

#### Caspase activities

Western blotting was used to measure the activities of caspase-9 and caspase-3. As shown in Fig. 6b, TPH/PTX nanomicelles significantly enhanced the activities of caspase-3 and caspase-9 in both A549 and A549/ADR cells. Interestingly, the expression of caspase-3 and caspase-9 in response to TPH/PTX nanomicelles was nearly equivalent in A549 and A549/ADR cells, suggesting that intrinsic apoptosis was activated by TPH/PTX nanomicelles.

#### Expression of Bcl-2 family proteins

Bax, as a proapoptotic member of the Bcl-2 family, functions as a regulator of apoptosis, while the anti-apoptotic members of the Bcl-2 family (such as Bcl-2) play a critical role in cell survival by interfering with the process of programmed cell death [28]. In the current study, the TPH/PTX nanomicelles enhanced proapoptotic protein (Bax) expression and reduced antiapoptotic protein (Bcl-2) expression in A549 and A549/ADR cells (Fig. 6b). TPH/PTX nanomicelles showed a positive outcome compared to the Taxol and TP/PTX nanomicelles, indicating that TPH/PTX nanomicelles could increase the apoptosis of drug-resistant A549/ADR cells by activating proapoptotic proteins and suppressing antiapoptotic proteins.

### In vivo imaging of drug-resistant cancer xenografts in mice

**Fig. 7:**
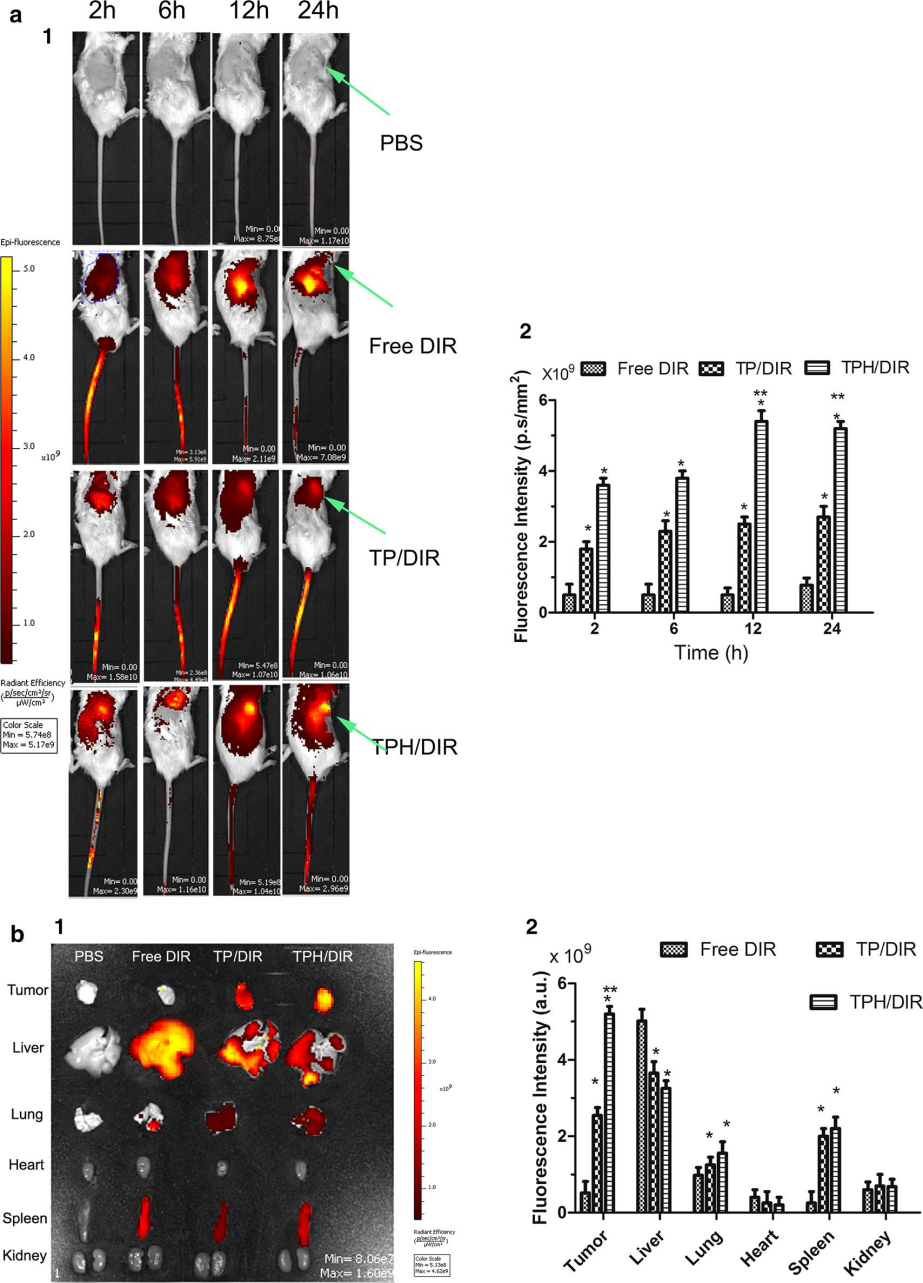
Tumor target ability of every formulations in 4T1/ADR cells xenografts in BALB/c mice after i.v. of free DIR, TP/DIR, TPH/DIR nanomicells. **a** In vivo real-time non-invasive whole-body imaging. **b** Ex vivo fluorescence of tumors and organs isolated from 4T1/ADR cells xenografts in BALB/c mice. (1) Image of the distribution of drugs in the body or tumors and organs. (2) Fluorescence intensity of image. Data are presented as the mean ± SD (n = 3). *P < 0.05 vs. free DIR; **P < 0.01 vs. TPH/DIR for 2 h, 6 h, respectively

The biodistribution of DIR was observed using an NIR reflection fluorescence imaging system and quantified by region-of-interest (ROI) analysis in drug-resistant breast cancer xenografts in mice. Figure 7a shows that only weak fluorescence at the tumor site was observed in the free DIR group until 24 h after injection. In contrast, at 6 h, TPH/DIR nanomicelles showed fairly strong fluorescence compared with the other groups, and then, the signals gradually became strong, possibly due to targeting of the CD44 receptor in the medium.

The major organs and the tumors were excised for ex vivo imaging at 24 h after injection. ROI analysis was used to quantitatively determine the fluorescence intensity. As shown in Fig. 7b, the TPH/DIR nanomicelle group revealed the strongest fluorescence signal in tumors, and its fluorescence intensity was 5.76-fold and 2.04-fold higher than that of free DIR and TP/DIR nanomicelle group, respectively.

### Anticancer efficacy in resistant human lung cancer xenografts

**Fig. 8:**
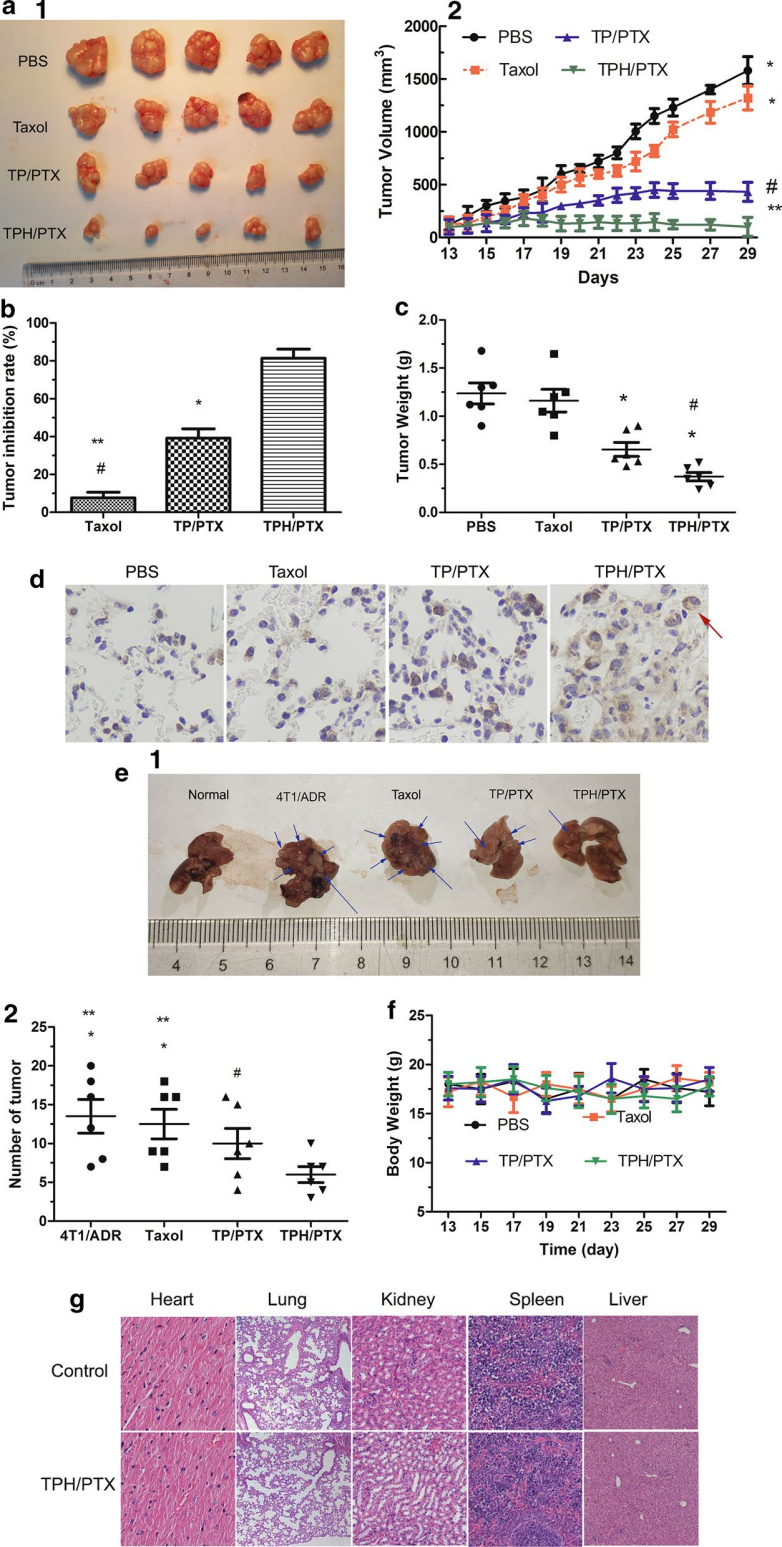
Anticancer efficacy and preliminary safety evaluation in resistant cancer xenografts. **a** Tumor images (1) and tumor growth inhibition graph (2) and tumor weight (**b**) for a murine model with A549/ADR xenografts after intravenous injection with the different formulations. **c** The tumor inhibitory rate (TIR). The TIR is calculated using the following equation: TIR (%) = [1 − X/Y] × 100%. X, the average weights of the tumors from the experimental groups; Y, the average weights of the tumors from control groups). **d** The expression of cytochrome C of the tumor tissues by immunohistochemistry assay. Images of lung cancer metastasis (**e1**) and tumor number (**e2**) for a murine model with 4T1/ADR xenografts after intravenous injection with the different formulations. Body weight (**f**) and H&E (**g**) stained organ slices from the A549/ADR-bearing nude mice treated with all formulations in vivo. The data are presented as the means ± standard deviations (n = 5); *P < 0.05, ^#^*P* < 0.001 compared with TPH/PTX nanomicells; **P < 0.05, compared with TP/PTX nanomicells

The analysis of different formulations with the same dose of PTX using resistant A549/ADR-xenografted nude mice revealed that the most significant antitumor activity was obtained with the TPH/PTX nanomicelles (Fig. 8a, b). On day 29, the mice treated with TPH/PTX nanomicelles achieved the highest tumor inhibition (81.7%), which was 8.09-fold and 2.12-fold higher than that of the Taxol and TP/PTX nanomicelle groups, respectively (Fig. 8c). The results of the immunohistochemical assay are shown in Fig. 8d. More apoptotic cells in the tumor were recorded in the TPH/PTX group than in all other groups, which showed no or very few apoptotic cells, further confirming the antitumor effect of TPH/PTX in resistant human lung cancer.

### Antitumor effects on lung metastasis in the drug-resistant breast cancer-bearing mouse models

As shown in Fig. 8e, 4T1/ADR tumor cells were given to Balb/c mice by intravenous injection at 10 days to a drug-resistant breast cancer-bearing mouse model with lung metastasis. Number of tumor from lung metastasis were significantly showed in the 4T1/ADR tumor mice. Tumor number with TPH/PTX nanomicells treatment significantly 3.54-fold or 2.01-fold less than Taxol or TP/PTX nanomicells. These data further support the results described above.

### Safety evaluation of nanomicelles in vivo

The body weights of the animals were monitored, as shown in Fig. 8f. The body weights of mice after administration of TPH/PTX nanomicelles were not significantly different from those after administration of other formulations. There was no evidence of noticeable organ damage by H&E staining (Fig. 8g). These results showed that TPH/PTX did not exhibit substantial cytotoxicity.

## Discussion

The clinical outcomes of new drug delivery systems (DDSs) are limited by the adverse effects of conventional cancer chemotherapy. Chemotherapeutic efficacy can be significantly improved through specific delivery of anticancer drugs to the site of the tumor. Given the increasing complicated pathogenesis of cancer, bifunctional DDSs are receiving increasing attention.

Given that apoptotic resistance is observed in many types of cancer cells [29], the ability to intervene by targeting apoptotic agents to mitochondria could enable the development of new anticancer strategies. TPP, as an effective mitochondria-targeting molecule, has been used as a targeting carrier [30–32] to facilitate selective accumulation of these DDSs in mitochondria. TPP, which consists of three phenyl groups, exhibits highly lipophilic properties and delocalization of the positive charges on phosphonium in three aromatic rings. Thus, TPP was conjugated to the amphipathic PF127 through an esterification reaction to produce TPP-PF127. Preliminary studies show that Pluronic molecules not only exhibit important biological activities, such as the general properties of the block copolymer micelles mentioned above, but also restore the sensitivity of MDR tumor cells to antineoplastic agents [19, 33].

To prolong the blood circulation time of TPP-PF127 with positive charge nanomicelles and avoid opsonization by plasma proteins, negatively charged HA was used to modify TPP-PF127 through ester bonds to form TPH polymers, and tumor cells were targeted via CD44 receptors, which are overexpressed in many cancer cells, resulting in increased therapeutic efficacy [26]. HAase, which is widely distributed in the acidic tumor extracellular matrix and lysosomes, can easily degrade HA to achieve efficient cellular uptake, lysosomal escape and mitochondrial targeting [34].

TPH/PTX nanomicelles exhibited excellent physical properties, such as, proper particle size, high encapsulation efficiencies, and a slightly positive polydispersity index (PDI) (Table 1) enable the TPH/PTX nanomicelles to be transported into tumor tissue by the enhanced permeability retention (EPR) effect. Compared with TP/PTX nanomicelles, the TPH/PTX nanomicelles were not very large, as observed by TEM. This result further demonstrated that the size of these nanomicelles was mainly attributed to the hydrophobic part [35]. In addition, after incubation with and without medium containing 10% FBS, the sizes of the three PTX-loaded nanomicelles were almost maintained for 25 h, indicating the excellent stability of the three nanomicelles.

During the initial 2 h, the delayed drug release (< 30%) would be beneficial for preventing rapid leakage during the process of delivery and would quickly increase the accumulation of the drug in the tumor masses. Due to the ester bond is pH sensitive [36, 37], the cumulative release of PTX from TPH/PTX at pH 4.5 is higher than those at pH 5.0 and 7.4, indicated that in acid solid tumor tissue, the drug could be released quickly, further achieved better anti-tumor activity in vivo.

In the cytotoxicity assay, Taxol, as a free small molecular drug, entered cancer cells as easy as ABC, thus excellent A549 cancer cells inhibition rate was observed in vitro anti-tumor activity assay. IC50 in the resistant A549/ADR with Taxol treatment, however, more 6.52-times than that in A549. TPH/PTX exhibited the strongest inhibitory effect on both A549 cells (Fig. 3a) and A549/ADR cells (Fig. 3b), compared with the other groups, and this effect was dose dependent and time dependent. The likely underlying mechanism is the rerouting of PTX to mitochondria. The results are supported by the data from the apoptosis assay.

Cells can engulf different molecules through a myriad of endocytic mechanisms [38]. Cellular uptake using various inhibitor assays showed that amiloride-treated cells internalized the TPH/C6 nanomicelles at significantly lower levels than the control cells. On the other hand, TPH/C6 nanomicelles penetrated into the cells through macropinocytosis, which was inhibited by amiloride pretreatment.

TPH/C6 nanomicelles accumulated in the acidic lysosomes after these nanomicelles entered the cancer cells, and lysosome escape of the TPH/C6 nanomicelles must occur before trafficking to mitochondria. Over 12 h, the merged signal changed in color from faint yellow (2 h) to stronger yellow (4 h), faint yellow (8 h), and Kelly green (12 h), indicating that TPH/C6 crossed the lysosomal pathway. In A549/ADR, the color of the merged signal was not changed for free C6, but TP/C6 and TPH/C6 showed significant changes. In particular, compared with A549, the color of the merged signal of TPH/C6 was nearly consistent with that in A549/ADR, indicating that TPH/C6 can effectively overcome drug resistance. It is possible that TPH/C6 nanomicelles were further degraded by HAase distributed in acidic lysosomes after internalization and that the positive charges were exposed, causing them to act as “proton sponges”. Proton absorbance by buffering with positively charged TPP prevents acidification of endosomal vesicles, thereby increasing the ATPase-mediated flux of protons and counter ions, which in turn leads to osmotic swelling, endosomal membrane rupture, and eventual leakage of the nanomicelles into the cytosol, making them accessible for mitochondrial uptake [39].

After lysosome escape, mitochondrial colocalization or increased mitochondrial uptake occurred due to the positive charge of TPP on TPH/C6. The fluorescence intensity of TPH/C6 at 12 h corresponds to that at 24 h, indicating effective inhibition of multiple mitochondrial targets. The drug content in the isolated mitochondria also indicated that the internalized nanomicelles in A549 and A549/ADR cells were not attached to the surfaces of the mitochondria but were further endocytosed by mitochondria.

Depolarization of the transmembrane potential (Δψm) is often induced by the opening of the MPTP. In addition, the loss of Δψm may not only result in cytochrome C release but also activate the apoptotic signaling pathway. In the present study, TPH/PTX significantly decreased the mitochondrial membrane potential (Δψm), caused the release of cytochrome C, activated the apoptotic caspases 9/3, increased the expression of proapoptotic Bax, and inhibited the expression of antiapoptotic Bcl-2, further indicating the involvement of mitochondrial signaling pathways in subsequent apoptosis. Interestingly, the expression of caspase-3, caspase-9, Bax and Bcl-2 in response to TPH/PTX was nearly equal between A549 and A549/ADR cells, suggesting that P-gp was overcome by TPH/PTX. These results showed that the apoptosis induced by TPH/PTX was mediated by mitochondria-dependent apoptotic pathways [40] and confirmed the mitochondria-targeting effect of TPH/PTX [41].

For up to 24 h, TP/PTX, and especially TPH/PTX exhibited strong fluorescence at the tumor location in vivo, indicating that HA coating prolonged the blood circulation of nanomicelles and increased the accumulation of the nanomicelles at tumoral sites via specific recognition by CD44 on cell surfaces [42, 43]. This result was further supported by ex vivo fluorescence Imaging.

Tumor volume, lung metastasis of drug-resistant breast cancer, TUNEL, and immunohistochemical assays showed that TPH/PTX exhibited the strongest tumor inhibitory effect. In summary, the following aspects can show the mechanism underlying the strong therapeutic efficacy of TPH/PTX on A549/ADR xenografts. First, HA, as a targeted ligand, can promote the uptake of TPH/PTX nanomicelles by bypassing drug efflux. Second, TPP conjugated with PF127, which had a low HLB value, was easily internalized into tumor cells. Finally, TPP, as a mitochondria-targeted ligand, can help TP/PTX enter mitochondria and overcome drug resistance by activating apoptosis.

## Conclusions

This study demonstrated the advantages of targeting the mitochondria of cancer cells to combat drug resistance and significantly enhanced the mitochondrial delivery of PTX through TPH/PTX nanomicelles. The specific uptake of these nanomicelles can be enhanced in drug-resistant cells via CD44 molecule-mediated endocytosis and by avoiding P-gp-mediated drug efflux. This result can facilitate mitochondrial targeting of the positively charged TPP medium to overcome the drug resistance of lung cancer cells. Mitochondrial delivery as a means of ‘‘repurposing’’ of FDA-approved drugs currently used in the clinic appears to be a worthwhile strategy to pursue for the development of new anticancer agents.

## Supplementary information

**Additional file 1: Figure S1.** Viability of A549 (a) and A549/ADR (b) cells cultured with PBS, TP, TPH, PTX-loaded nanomicelles in comparison with that of Taxol at the same PTX dose for 48 h. All data are presented as the means ± standard deviations (n = 3). *P ≤ 0.05 with TPH/PTX.

## Data Availability

All data generated or analyzed during this study are included in this published article (and its additional information files).

## Abbreviations

MDR: multiple-drug resistance
P-gp: P-glycoprotein
PF127: Pluronic F127
TPH: TPP-Pluronic F127-hyaluronic acid (HA)
TP: TPP-Pluronic F127
HAase: hyaluronidase
HLB: hydrophile–lipophile balance
DCC: *N*,*N*′-dicyclohexylcarbodiimide
DMAP: 4-dimethylaminopyridine
H&E: hematoxylin and eosin
TEM: transmission electron microscopy
C6: coumarine-6
FBS: fetal bovine serum
DCM: dichloromethane
DLC: drug-loading capacity
DLE: drug-loading efficiency
ROI: region-of-interest
MOMP: mitochondrial outer membrane permeabilization
DDSs: drug delivery systems
JC1: 5,5ʹ,6,6ʹ-tetrachloro-1,1′,3,3′-tetraethylbenzimidazolylcarbocyanine iodide
