# Supplementary material for: Tumor- and mitochondria-targeted nanoparticles eradicate drug resistant lung cancer through mitochondrial pathway of apoptosis
Source: J Nanobiotechnology. 2020 Jan 9;18:8. doi: 10.1186/s12951-019-0562-3 (PMC6950814; doi:10.1186/s12951-019-0562-3)
Supplement: Supplementary file 1 — Additional file 1: Figure S1. Viability of A549 (a) and A549/ADR (b) cells cultured with PBS, TP, TPH, PTX-loaded nanomicelles in comparison with that of Taxol at the same PTX dose for 48 h. All data are presented as the means ± standard deviations (n = 3). *P ≤ 0.05 with TPH/PTX. [file 12951_2019_562_MOESM1_ESM.docx]

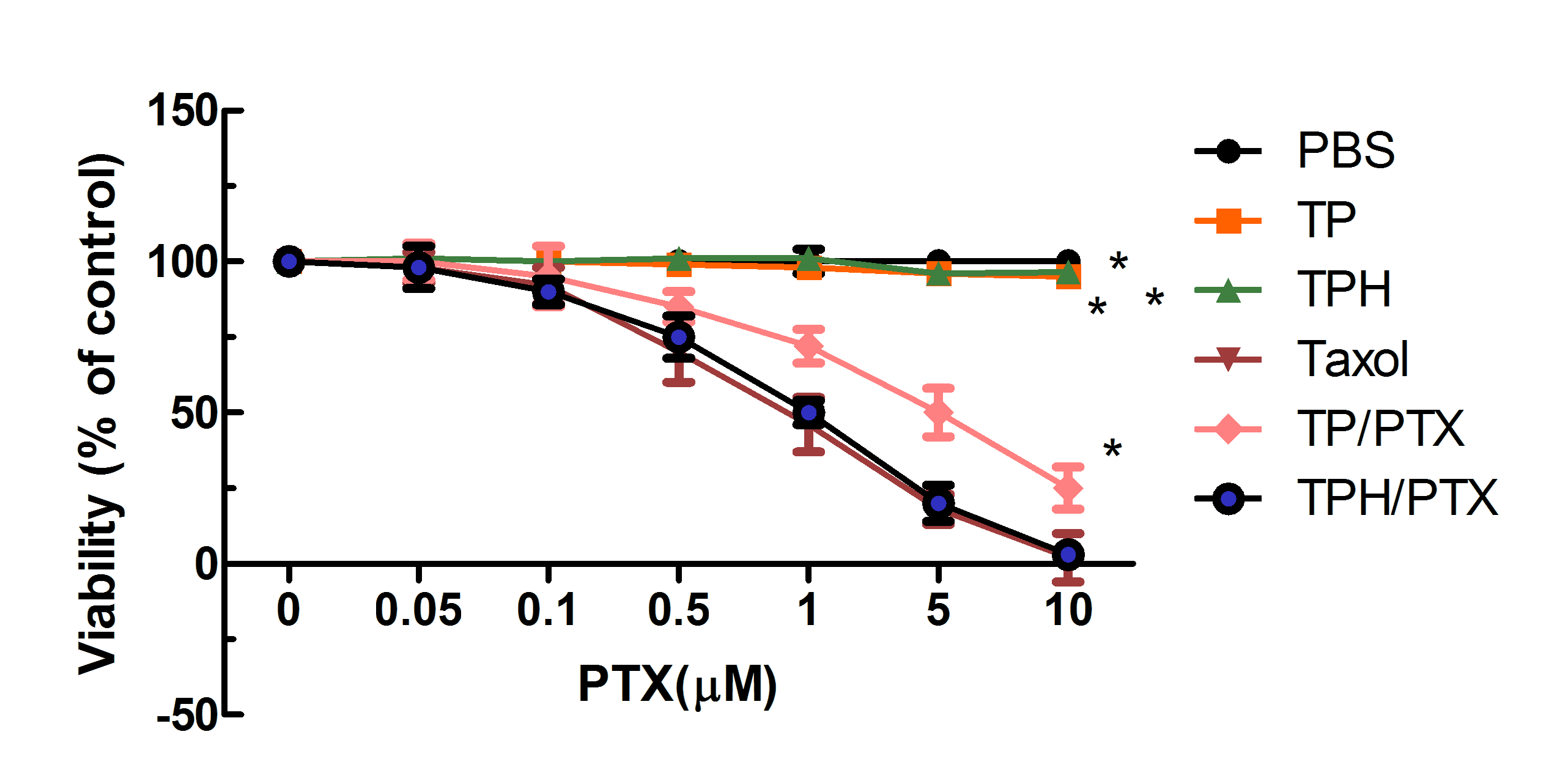


a


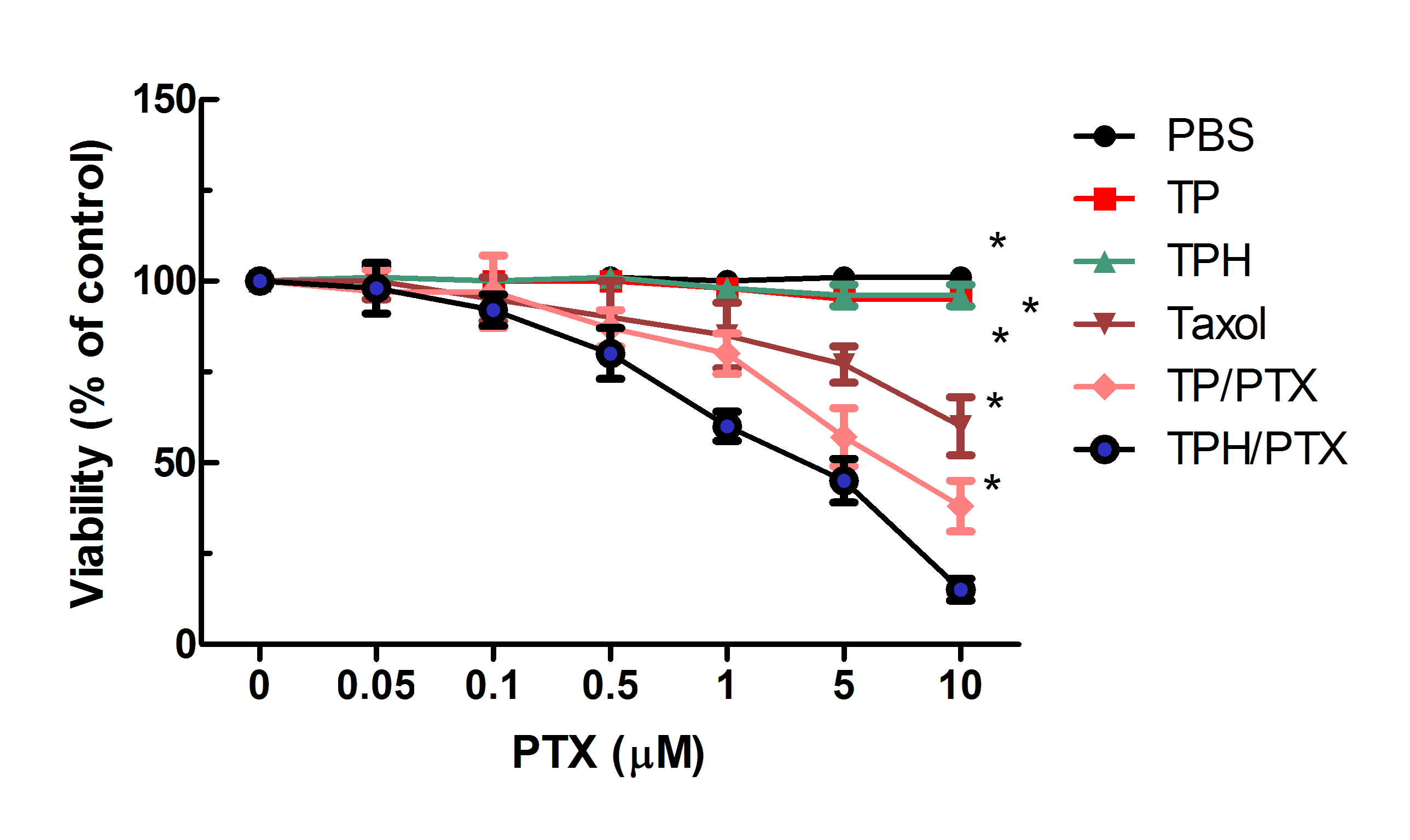


b

Figure Viability of A549 (a) and A549/ADR (b) cells cultured with PBS, TP, TPH, PTX-loaded nanomicelles in comparison with that of Taxol at the same PTX dose for 48 h. All data are presented as the means ± standard deviations (n = 3). *P≤0.05 with TPH/PTX.
